# Case series: Cariprazine in early-onset schizophrenia

**DOI:** 10.3389/fpsyt.2023.1155518

**Published:** 2023-04-14

**Authors:** Elena Ivanova, Desislava Maslinkova, Nadia Polnareva, Vihra Milanova

**Affiliations:** ^1^Clinic of Child Psychiatry “St. Nicholas”, University Hospital “Alexandrovska”, Sofia, Bulgaria; ^2^Department of Psychiatry and Medical Psychology, Medical University - Sofia, Sofia, Bulgaria; ^3^Clinic of Psychiatry, University Hospital “Alexandrovska”, Sofia, Bulgaria

**Keywords:** negative symptoms, Cariprazine, novel atypical antipsychotics, tolerability, general and social functioning, early-onset/adolescent schizophrenia

## Abstract

**Introduction:**

Negative symptoms are part of the clinical manifestations of schizophrenia and their presence is associated with a poorer prognosis, significantly limited vocational opportunities, impaired quality of life and social functioning. In the clinical practice, treatment of negative symptoms in patients with schizophrenia, is a challenge. Cariprazine is a novel partial agonist of D3 and D2 receptors, and shows a high affinity for D3, with good tolerability, good response to schizophrenic symptoms and limited side effects. We present two cases of young patients with predominantly negative symptoms during treatment with an atypical antipsychotic, administered in a stable dose and therapeutic range, and for at least 4 weeks prior to the Cariprazine switch.

**Methods:**

Two patients (men aged 21 and 22) with schizophrenia, exhibiting predominantly negative symptoms, are presented. Their diagnosis was based on, DSM-5 criteria (295.10).

Patients were treated with Cariprazine at a daily dose of 4.5 mg. They were followed for a period of 18 months and assessed with Positive and Negative Syndrome Scale (PANSS), Global Assessment of Functioning (GAF) and Clinical Global Impression-Severity (CGI-S), at the fourth week of initiation of treatment with Cariprazine, at 6 months, at 12 months and at 18 months. Their mean initial value was 75.5 on PANSS, 4.0 on CGI-S, and 52.5 on GAF. Both patients were treated with stable doses of atypical antipsychotic–Risperidone at a daily dose of 4,5 mg. Cross-titration to Cariprazine was initiated, from 1.5 mg daily dose up to 4,5 mg daily dose, during a period of 2 weeks.

**Results:**

After 18 months of treatment with Cariprazine at a daily dose of 4.5 mg, the following results were reported: mean value was 57.5 on PANSS, 3.0 on CGI-S, and 74.5 on GAF. The overall PANSS mean score decreased by 23.8%, the CGI-S mean score improved by 25% and the mean GAF score increased by 29.5%. The positive PANSS subscale score decreased minimally, from 20 to 16, while for the negative subscale the improvement was 29.8%.

Cariprazine was well tolerated by patients and no side effects were observed from it during therapy.

**Discussion:**

After 18 months Cariprazine succeeded in improving negative symptoms, global functioning, and global clinical impression. In young schizophrenic patients with a predominance of negative symptoms, the cariprazine may be a successful alternative.

## Introduction

Schizophrenia exists on a spectrum of psychotic disorders. It is manifested in clinical polymorphism and a diverse combination of symptoms in structure, course, and outcome. In this mental disorder, there are also age-specific features in its clinical presentation. Schizophrenia is a multifactorial illness in which structural and functional brain abnormalities, dysfunctions in neurotransmitter systems, genetic predisposition, and other factors have been studied.

The onset of schizophrenia at a young age affects personal and school achievement, social functioning, career choices, and family and peer relationships. Patients before the age of 18 with first psychotic episode is about 11–18% (1). Schizophrenia under 18 is divided in: early-onset schizophrenia (between the ages of 13 and 17) and very–early- onset schizophrenia (before the age of 12 years) (2).

Schizophrenic symptoms are: positive (delusions, hallucinations, disorganized speech and behavior) and negative (blunted affect, alogia, asociality, anhedonia, and avolition) (3, 4), affective (depression, anxiety, hypomania) (5) and cognitive symptoms (impaired attention, difficulties in verbal fluency and memory, poor working memory and deficits in executive functioning) (6).

Negative symptoms are impairments that fall within the clinical manifestations of schizophrenia. Both the concept of negative symptoms and the development of their effective treatment have a history.

The term “negative symptoms” describes the absence or impairment of functions related to interests and motivation, emotional and verbal expression and age appropriate behavior. The negative symptom domain consists of the following constructs: blunted affect, alogia (reduction in quantity of words spoken), avolition (reduced goal-directed activity due to decreased motivation), asociality, and anhedonia (reduced experience of pleasure).

The National Institute of Mental Health (NIMH)-MATRICS (Measurement and Treatment Research to Improve Cognition in Schizophrenia) Consensus Statement on Negative Symptoms provided a possible model for proceeding in the area of negative symptoms (7).

According to the authors (8), negative symptoms and cognitive impairment represent separate domains. Targets for therapy are the negative symptoms that imply a loss or diminution of normal functions and/or a decrease in the quality of life that can be recognized by psychiatrists and relatives. Persistent and clinically significant negative symptoms are a therapeutic challenge.

Longitudinal studies (9, 10) can provide information on their persistence. Negative symptoms are significant parts of the disease process. The same authors point out that those patients with primary symptoms represent about 20–25% of the patients included in the clinical samples. In order to report on the effectiveness of the treatment, follow-up should be substantially longer (in the range of 6 months).

According to Kirkpatrick et al. (11) the treatment first shown to be effective for persistent negative symptoms may not be effective for primary negative symptoms (11).

First-generation antipsychotics (FGAs) and second-generation antipsychotics (SGAs) have relatively similar effectiveness, but SGAs are reported to be better tolerated by patients because they do not have the side effects of FGAs, such as extrapyramidal symptoms or tardive dyskinesia (12).

The metabolic side-effects that have been reported in the course of SGA treatment are weight gain and diabetes, leading to increased risk of cardiovascular complications (13).

The antipsychotics with a D2 receptor antagonism profile are effective in controlling positive symptoms, but they have less impact on negative symptoms, with the latter significantly affecting functional outcomes (14). Cariprazine, Aripiprazole, and other newer SGAs are dopamine D2 receptor partial agonists. They have the advantage of causing less metabolic side-effects than other SGAs, while potentially improving negative symptoms and cognitive functioning (15).

The partial agonists have the feature to elicit different activity depending on the environment: they block receptors in the presence of agonists with higher intrinsic activity, but they act as agonists themselves. This feature of partial D2 agonists determines the improvement of psychotic symptoms, while avoiding side effects (16).

Cariprazine is a medication that has the following receptor profile: partial agonist of dopamine D3 and D2 (with greater affinity for D3 dopamine receptors) and serotonin 5-HT1A receptors; an antagonist of 5-HT2B, 5-HT2A and histamine receptors–H1. Cariprazine has low affinity for serotonin 5-HT2C receptors and adrenergic α1 receptors. The drug shows almost no affinity for cholinergic muscarinic receptors. Cariprazine has good tolerability, which is probably also due to the poorly manifested anticholinergic, antiadrenergic, antihistaminergic and metabolic effects, extrapyramidal symptomatology, increase in prolactin values (17).

Cariprazine is unique because of its *in vivo* binding to D3 receptors (18).

The results of a meta-analysis of 32 antipsychotics conducted by Huhn et al. (19) showed that only few had partial effects against negative symptoms: Clozapine, Amisulpride, Olanzapine, and Risperidone. The authors have indicated as one of the exclusionary criteria the presence of predominant negative symptoms (19).

Nemeth et al. (20) compared two groups of patients with predominant negative symptoms, one undergoing treatment with Cariprazine (daily dose of 3 to 6 mg) and the other with Risperidone (daily dose of 3 to 6 mg). The patients treated with Cariprazine had better therapeutic effect versus negative symptoms (20). So, for patients with predominant negative symptoms Cariprazine is а suitable therapeutic alternative.

According to Fagiolini et al. (21), the medication of choice for patients with a first psychotic episode, with predominantly negative symptoms, is Carpiprazine (21).

In this article, we use terms “persistent,” “predominant,” and “prominent” for describing negative symptoms.

According to Buchanan (22), persistent negative symptoms include clinically stable patients whose negative symptoms persist with adequate antipsychotic drug treatment.” The clinical assessment of persistent negative symptoms is based on cross-sectional and longitudinal evaluation of negative symptoms (22).

Persistent negative symptoms are defined as „the presence of at least one negative symptom of moderate or higher severity, not confounded by depression or parkinsonism, at baseline and after 1 year of treatment” (23).

Patients with “prominent negative symptoms” are characterized as patients with a high degree of negative symptoms, while the definition of predominant negative symptoms includes the additional criteria of “no-to-little positive symptoms” (24).

Persistent, predominant and prominent negative symptoms corresponds to a certain score of positive and negative PANSS subscale items.

In this case series we present two young patients (at the age of 21 and 22) with long prodromal periods from the initial manifestation of symptoms to the diagnosis and 18 months of follow-up of negative symptoms.

## Method of study

The patients were assessed with Positive and Negative Syndrome Scale (PANSS) (25), Global Assessment of Functioning (GAF) (26) and Clinical Global Impression – Severity scale (CGI-S) (27) at the fourth week of initiation of treatment with Cariprazine, at 6 months, at 12 months, and at 18 months.

## Case presentation

### Patient 1

The patient was a 21-year-old man with schizophrenia. He did not have family history of mental disorders. His mental problems began at the age of 14. After a consultation with a psychiatrist he was diagnosed with obsessive–compulsive disorder (OCD) in DSM-5 (300.3) (28), and was prescribed therapy with Sertraline at an initial dose of 25 mg in the morning. The dosage was gradually increased to 150 mg in the morning. Compulsive control was achieved. The patient had maintenance therapy at the same dose for a period of about two and a half years. The patient himself discontinued his therapy.

At the age of 17, his parents reported changes in his behavior–he isolated himself, had no friends, significantly lowered his school performance, and had difficulties concentrating. During the last school year, he continued to struggle in school, had difficulty even getting help with his schoolwork, and showed no interest in previous activities. At the age of 18, he was consulted by a psychiatrist in regards to the following complaints. The patient shared that his classmates continued to influence him from a distance. His parents sometimes heard him talking to himself in his room. The patient isolated himself. He neglected his appearance. The patient refused to watch films on television, could not concentrate and was quickly losing interest. The night sleep lasted 4 to 5 h, with frequent awakenings during the night.

After the psychiatric consultation at the age of 18, he was diagnosed with schizophrenia according to DSM–5 criteria (295.10). A structured psychiatric assessment interview was conducted.

The initial psychiatric examination detected residual positive symptoms (mild suspiciousness, conceptual disorganization), negative symptoms (blunted affect, alogia, asociality, anhedonia, and avolition), as well as general symptoms (insomnia, poor attention, low memory performance, social withdrawal and motor retardation). Laboratory tests, somatic and neurological examinations did not identify any comorbidities during the initial visit.

The patient was prescribed Risperidone and the dose was gradually increased to 4.5 mg daily. After reduction of the psychotic symptoms (with residual delusions for influence), a slight predominance of negative symptoms was reported. Patient had medication side effects (extrapyramidal symptoms) during Risperidone treatment and therefore he was switched to Cariprazine treatment at an initial dose of 1.5 mg with a dose increase up to 4.5 mg in the morning on the 14^th^ day. On the second week of taking Cariparzine, the dose of Risperidone was reduced and on the third week Risperidone was discontinued. The patient passed the crossover period calmly.

At the initial assessment PANSS total score was 77 (31 points on the negative subscale and 21 points on the positive subscale), CGI-S value was 4, and GAF score was 54.

After 18 months of stable dose intake of Cariprazine–4.5 mg daily, PANSS total score decreased to 58, with the negative scale showing a value of 21, CGI-S score decreased to 3, and GAF score increased to 78. The positive PANSS score decreased minimally – from 21 to 16. This patient reported no adverse events during the 18 months of the 4.5 mg daily Cariprazine regimen. The patient showed improvement in social contacts and participated for 3–4 h in daily activities at the family company, where he archived documents.

### Patient 2

The patient was a 22-year-old man with schizophrenia. He had a family history of schizophrenia–his father also had the disorder.

According to his mother, around the age of 14–15 years, he became more withdrawn, had no friends, did not communicate with anyone and often isolated himself. The patient had difficulty concentrating, which made it difficult to learn.

His parents divorced. Despite the divorce, the children (the patient and his younger sister) maintained regular contact with their father. The patient’s mother did not consult a psychiatrist during this period.

At the age of 16, the patient witnessed an accident in which his father drowned. Afterwards, there was a change in his mental state: he became withdrawn and silent, his sleep at night was disturbed, he isolated himself in his room, and he did not communicate with others.

A few weeks after the accident, by the start of the school year, he refused to attend school. The mother sought help from a psychologist. The patient, after a consultation with a psychologist, was referred to a psychiatric examination. The patient told the psychiatrist about painful moments in which he relived what had happened to his father. He was diagnosed with Post Traumatic Stress Disorder (PTSD) (309.81) and met the criteria for DSM-5. He was prescribed Diazepam–5 mg in the evening for 10 days, in order to improve night sleep and reduce tension. He was referred to psychotherapy. The patient took only a few nights of his prescribed therapy, discontinued the medication himself, and refused to see a psychologist.

In the following years, the patient underwent significant changes in his behavior: he did not leave his room, stayed in the dark, and drew strange geometric shapes on his body to “protect” his relatives. He placed various objects and food in water, spilling water on the floor of the home to “purify” the home. He did not take care of his appearance. Night sleep was disturbed. He smoked up to 30 cigarettes a day.

At the age of 18, after two sleepless nights, he became verbally aggressive toward his mother. The patient was consulted urgently by a psychiatrist and diagnosed with schizophrenia – the criteria for DSM–5 (295.10). An additional psychiatric interview was conducted.

He was hospitalized and treated with Haloperidol–5 mg daily (intramuscular) to control his aggressive behavior motivated by psychotic reasons. On day 5 of the hospitalization, a switch in medication seemed necessary due to side effects (extrapyramidal symptoms) of Haloperidol. The patient was prescribed Risperidone with dosage regimen 4.5 mg/day. After failing to achieve complete remission of the delusional symptoms and aggressive behavior due to psychotic exacerbations Cariprazine was added to the therapy–initially 1.5 mg daily, on the 14th day the dose was increased to 4.5 mg daily, and on the third week Risperidone was discontinued.

During the initial psychiatric examination, the patient presented with: fragmentary persecutory delusions without significant behavioral impact, mild suspiciousness and conceptual disorganization and predominant negative symptoms, including especially blunted affect, alogia, asociality, anhedonia, and avolition, as well as general symptoms–insomnia, social withdrawal, poor attention and low memory performance.

Laboratory tests, somatic and neurological examinations did not identify comorbidities during the initial visit.

His baseline PANSS total score was 74 (negative subscale score of 26 and positive subscale score of 19), CGI-S score of 4, and GAF score–51. The patient went through the titration period without any significant dynamics in his state. The intake of Cariprazine was not accompanied by side effects.

After 18 months of stable dose intake, PANSS total score decreased to 57, with the negative subscale showing a value of 19, CGI-S score decreased to 3, and GAF score increased to 71. The positive subscale PANSS score decreased minimally, from 19 to 16. This patient reported no adverse events during the 18 months of the 4,5 mg daily Cariprazine regimen.

In the course of the treatment, he improved communication with his relatives, started to take care of his personal hygiene and went for walks.

The following differential diagnoses were discussed for both patients: Intellectual disability, Unipolar major depression with psychotic features, Schizoaffective disorder, no evidence of substance use or abuse,. Other mental disorders were ruled out in the initial assessment based on an interview using the Mini-International Neuropsychiatric Interview (MINI) (29).

**Figure 1:**
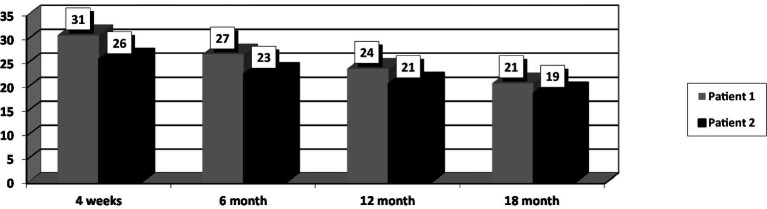
Dynamics of PANSS negative subscale values in the course of therapy with Cariprazine.

**Figure 2:**
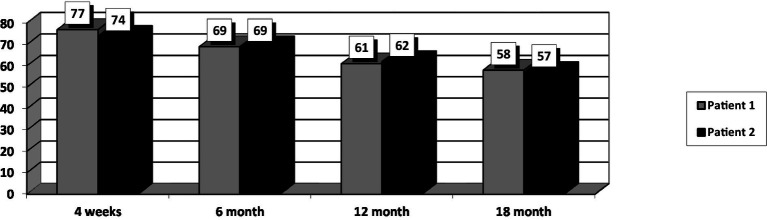
Dynamics of the overall PANSS mean score in the course of therapy with Cariprazine.

**Figure 3:**
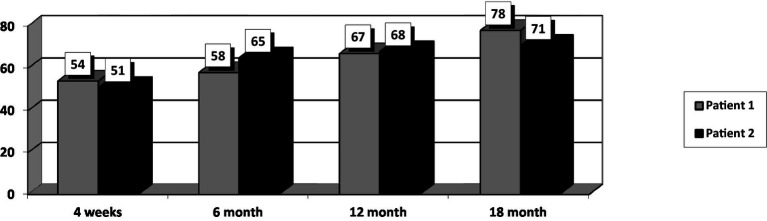
Dynamics of the mean GAF scores in the course of therapy with Cariprazine.

After 18 months of Cariprazine – at a 4.5 mg daily dose, the positive subscale of PANSS showed relatively no significant change, but the negative subscale mean score decreased by 29.8% (Figure 1). The overall PANSS mean score decreased by 23.8% (Figure 2), CGI-S mean score improved by 25%, and the mean GAF score increased by 29.5% (Figure 3).

The summary results of the follow-up are presented in Table 1, which allows a comparison of the initial results and of the results obtained after 18 months of follow-up.

**Table 1:** Presentation of the results–baseline and after18 months with Cariprazine.

	Patient 1	Patient 2
Рrevious medication and dose	Risperidone – 4,5 mg/day	Risperidone – 4,5 mg/day
Cross-titration to Cariprazine	14 days	14 days
PANSS total score- initial visit	77	74
PANSS total score- final visit	58	57
PANSS Negative scale score- initial visit	31	26
PANSS Negative scale score- final visit	21	19
PANSS Positive scale score- initial visit	21	19
PANSS positive scale score- final visit	16	16
CGI-S initial score	4	4
CGI-S final score	3	3
GAF initial score	54	51
GAF final score	78	71
Adverse events throughout the 18 months follow-up period	None	None

## Discussion

Positive, negative, affective and cognitive symptoms may be present in the clinical manifestations of psychotic disorders and they are present in varying degrees. According to researchers, the pharmacological profile of Cariprazine makes it a multimodal antipsychotic that can cover a significant spectrum of psychotic symptoms (20, 30).

The presented patients were of young age (21 and 22 years). What is interesting in both cases is the presence of mental disorders (OCD and PTSD) preceding the manifestations of psychosis, the long prodromal phase, the presence of negative symptoms (blunted affect, alogia, asociality, anhedonia, and avolition) and general symptoms – insomnia, poor attention, low memory efficiency and social withdrawal.

According to researchers, there is a high comorbidity between OCD and schizophrenia, with the following associations: male gender, age of onset of OCD before 20 years, and prescription of antipsychotics (31).

Patients diagnosed with OCD are at risk for developing earlier onset of psychosis, more severe psychopathology, manifestations of social dysfunction, and higher rates of hospitalization (32–34).

Based on data from the Swedish National Register, patients diagnosed with OCD had a 2.7-times higher risk of a subsequent diagnosis of schizophrenia compared to those without OCD (35). Similar results were obtained on Danish register, prior diagnosis of OCD was associated with an increased risk of developing schizophrenia and schizophrenia spectrum disorders later in life (36).

After a traumatic stress disorder there was a significantly increased risk of schizophrenia, schizophrenia spectrum disorder and bipolar disorder. Risks were highest in the first year after diagnosis of the traumatic stress disorder and remained significantly elevated after more than 5 years (37). A meta-analysis found a mean prevalence rate of PTSD of 12.4% in patients with schizophrenia (38).

Traumatic experiences in childhood have been associated with an increased risk of developing schizophrenia (39).

In this case, after 18 months of treatment with Cariprazine, there was a 29.8% reduction in negative symptoms, 29.5% improvement in global functioning, and 25% improvement in clinical global impression.

There are differences between the negative symptoms reported by PANSS and those listed in the Consensus Statement on Negative Symptoms.

PANSS structure includes five symptom complexes defined by the scale (blunted affect, alogia, avolition, anhedonia, and attention impairment) (40).

Blunted affect is included in commonly used negative symptom rating scales, such as PANSS. In PANSS, the focus of the assessment is on facial expressions and communicative gestures. Alogia is defined as a reduction in the quantity of speech and in its spontaneous elaboration. In PANSS, the symptom is named “lack of spontaneity and flow of conversation” and described as a decrease in the normal flow of communication associated with apathy, avolition, defensiveness or cognitive impairment. The assessment of anhedonia is not included in the PANSS negative subscale. The assessment of asociality mostly relies on the subject’s behaviour according to PANSS. Emotional withdrawal in PANSS, actually refers to avolition. The evaluation of alogia and blunted affect provided by PANSS is based on different items, some of which are not relevant to the negative symptom domain (poverty of speech content, inappropriate affect). The assessment of avolition and asociality also varies greatly. Anhedonia is not rated in PANSS (3).

Both patients had improvement of the following negative symptoms. Asociality was reduced (it refers to withdrawal or lack of desire for social contact). Improvement was also reported for blunted affect, anhedonia, alogia and avolition.

The results we obtained: good therapeutic effect against negative symptoms and improved overall functioning, were confirmed by another author Vasiliu (41), who followed three cases of schizophrenic patients with predominantly negative symptoms who were treated with Cariprazine for a period of 12 weeks. The same author estimated the dynamics in the state using: PANSS, GAF and CGI-S. The results obtained were: reduction of negative symptoms, improvement in social functioning and good tolerability of the medication (41).

In conclusion, the factors that influence the effectiveness of drug therapy are: the age of disease onset, the time interval between the initial onset of symptoms and the timely initiation of drug therapy, the clinical symptoms (positive and/or negative symptoms), family history of mental disorders and others psychiatric disorders preceding schizophrenia.

The limitations of the cases presented are: the very small number of patients and scales used for evaluation, which may have prevented the observation of other, longer-term effects of Cariprazine administration, and the large time interval of follow-up (6 months). The tolerability of Cariprazine was assessed based on information provided by the patient and the data was obtained during each examination without using a structured approach.

PANSS was used to assess negative symptoms over a 6-month period. Psychometric studies provide information about the dynamics of negative symptoms, but they also have a number of limitations. The Consensus Statement on Negative Symptoms postulates the need for development of an instrument that includes all of the 5 domains: blunted affect, alogia, asociality, anhedonia and avolition (2).

The patients’ conditions were stable and did not require hospitalization during follow-up. The follow-up of these two cases reveals the potential for a better prognosis in young patients with early-onset/adolescent schizophrenia undergoing treatment with Cariprazine.

### Patient perspective

“I had no strength, no energy. I felt like things were pounding in my head, it was hard to even think. I was distracted…. I had fears….. I can already do different things to help at home.” (Patient 1).

“I stayed only in the apartment and did not want to go out, I was afraid. … I talk to my mother and my fears are less. I already have the strength to take care of some things.” (Patient 2).

## Data availability statement

The original contributions presented in the study are included in the article/supplementary material, further inquiries can be directed to the corresponding author.

## Ethics statement

Ethical review and approval was not required for the study on human participants with the local institutional requirements. The patients provided their written informed consent to participate in this study. Written informed consent was obtained from the participant/patient(s) for the publication of this case report.

## Author contributions

All authors listed have made a substantial, direct, and intellectual contribution to the work and approved it for publication.

## Funding

The authors declare that this study received funding from Gedeon Richter Plc. The funder was not involved in the study design, collection, analysis, interpretation of data, the writing of this article, or the decision to submit it for publication.

## Conflict of interest

The authors declare that the research was conducted in the absence of any commercial or financial relationships that could be construed as a potential conflict of interest.

## Publisher’s note

All claims expressed in this article are solely those of the authors and do not necessarily represent those of their affiliated organizations, or those of the publisher, the editors and the reviewers. Any product that may be evaluated in this article, or claim that may be made by its manufacturer, is not guaranteed or endorsed by the publisher.
